# Causal influences of osteoarthritis on COVID-19: a Mendelian randomization study

**DOI:** 10.3389/fmed.2023.1287043

**Published:** 2023-10-31

**Authors:** Li Fu, Ancha Baranova, Hongbao Cao, Fuquan Zhang

**Affiliations:** ^1^Department of Psychiatry, The Affiliated Brain Hospital of Nanjing Medical University, Nanjing, China; ^2^School of Systems Biology, George Mason University, Manassas, VA, United States; ^3^Research Centre for Medical Genetics, Moscow, Russia; ^4^Institute of Neuropsychiatry, The Affiliated Brain Hospital of Nanjing Medical University, Nanjing, China

**Keywords:** osteoarthritis, COVID-19, Mendelian randomization, GWAS, ACE

## Abstract

**Objective:**

Although observational and genetic studies have indicated a correlation between OA and COVID-19, it remains uncertain whether osteoarthritis (OA) contributes to the severity of COVID-19. Here, we aimed to investigate the potential causal links between the two.

**Methods:**

In this study, we conducted Mendelian randomization (MR) analysis to investigate whether there is a potential causal connection between OA and COVID-19 outcomes. The analysis utilized publicly available GWAS summary datasets, incorporating data on OA (*N* = 455,221), SARS-CoV-2 infection (*N* = 2,597,856), hospitalized COVID-19 (*N* = 2,095,324), and critical COVID-19 (*N* = 1,086,211). Additionally, we performed a literature analysis to establish a molecular network connecting OA and COVID-19.

**Results:**

The MR analysis showed causal effects of OA on hospitalized COVID-19 (OR: 1.21, 95% CI: 1.02–1.43, *p* = 0.026) and critical COVID-19 (OR: 1.35, 95% CI: 1.09–1.68, *p* = 0.006) but not on SARS-CoV-2 infection as such (OR: 1.00, 95% CI: 0.92–1.08, *p* = 0.969). Moreover, the literature-based pathway analysis uncovered a set of specific genes, such as *CALCA*, *ACE*, *SIRT1*, *TNF*, *IL6*, *CCL2*, and others, that were found to mediate the association between OA and COVID-19.

**Conclusion:**

Our findings indicate that OA elevates the risk of severe COVID-19. Therefore, larger efforts should be made in the prevention of COVID-19 in OA patients.

## Introduction

Ever since the onset of the COVID-19 pandemic, SARS-CoV-2 has emerged as a worldwide peril to public health. This virus, originally considered a respiratory infection, also damages the central nervous system (1–5), cardiovascular system (6), and various other tissues and organs (7). Furthermore, to date, numerous factors have been recognized to increase susceptibility to COVID-19, notably chronic conditions such as diabetes, hypertension, and others (8–12). Despite the decrease in the prevalence of COVID-19, it continues to present a health challenge to the population.

Osteoarthritis (OA) is one of the most common chronic musculoskeletal diseases in the world, impairing the quality of life for more than 10% of elderly individuals over 60 years old and 40% of those over 70 years old (13, 14). The primary pathological characteristic of OA is synovitis, with the damaged synovium secreting a variety of cytokines and chemokines that maintain inflammation and promote both cartilage degeneration and subchondral bone changes (15). In this case, patients with OA often experience chronic concomitant diseases such as cardiovascular diseases and systemic inflammation (15). These comorbidities may further reduce resistance and undermine health.

Epidemiological studies have shown that rheumatoid arthritis increases the risk of adverse outcomes of COVID-19 (16, 17). In the case of OA, it was found that OA displays positive genetic correlations with COVID-19 traits, and both conditions share certain chromosomal loci and particular genes. However, Mendelian randomization (MR) analysis of the same datasets did not substantiate the causal genetic relationships observed (18). Consequently, we endeavored to verify the causality between OA and the vulnerability and severity of COVID-19 with larger datasets.

Mendelian randomization (MR) analysis infers causal relationships between exposure factors and disease outcomes by taking advantage of the random combinations of genetic variants during meiosis and utilizing exposure-related genetic variants as instrumental variables (IVs) (19, 20). Due to its reliance on the random distribution of alleles, MR analysis is typically less prone to common confounding factors, such as lifestyle influences (21). Recently, the MR design has been widely utilized to detect causal relationships between diseases (22, 23). Here, we used summary GWAS datasets to perform MR analysis of the potentially causal relationships between OA and COVID-19. Additionally, we established the molecular pathway by employing knowledge-based analysis to gain deeper insights into this association.

## Materials and methods

### GWAS datasets and population

In this research, causal relationships were estimated using publicly accessible GWAS summary data. To ensure homogeneity in the population, the study participants were exclusively of European ancestry. The datasets of COVID-19 were sourced from the COVID-19 Host Genetics Initiative (HGI) GWAS, with the exclusion of 23 and Me data. The datasets covered outcomes of SARS-CoV-2 infection, hospitalized COVID-19, and critical COVID-19 (more information is available in Table 1) (24). In this study, we collectively described the cases of hospitalized COVID-19 and critical COVID-19 as “severe COVID-19” (10). The GWAS dataset of OA originated from UKB, consisting of knee osteoarthritis, hip osteoarthritis, knee and/or hip osteoarthritis, and any osteoarthritis phenotypes (detailed data in Table 1) (14).

**Table 1:** Information on the GWAS summary datasets.

Dataset	N-case	N-control	Population	URL
Osteoarthritis	77,052	378,169	European	https://ega-archive.org/
SARS-CoV-2 infection	122,616	2,475,240	European	https://www.covid19hg.org/results/r7/
Hospitalized COVID-19	32,519	2,062,805	European	https://www.covid19hg.org/results/r7/
Critical COVID-19	13,769	1,072,442	European	https://www.covid19hg.org/results/r7/

### MR analysis

**Figure 1:**
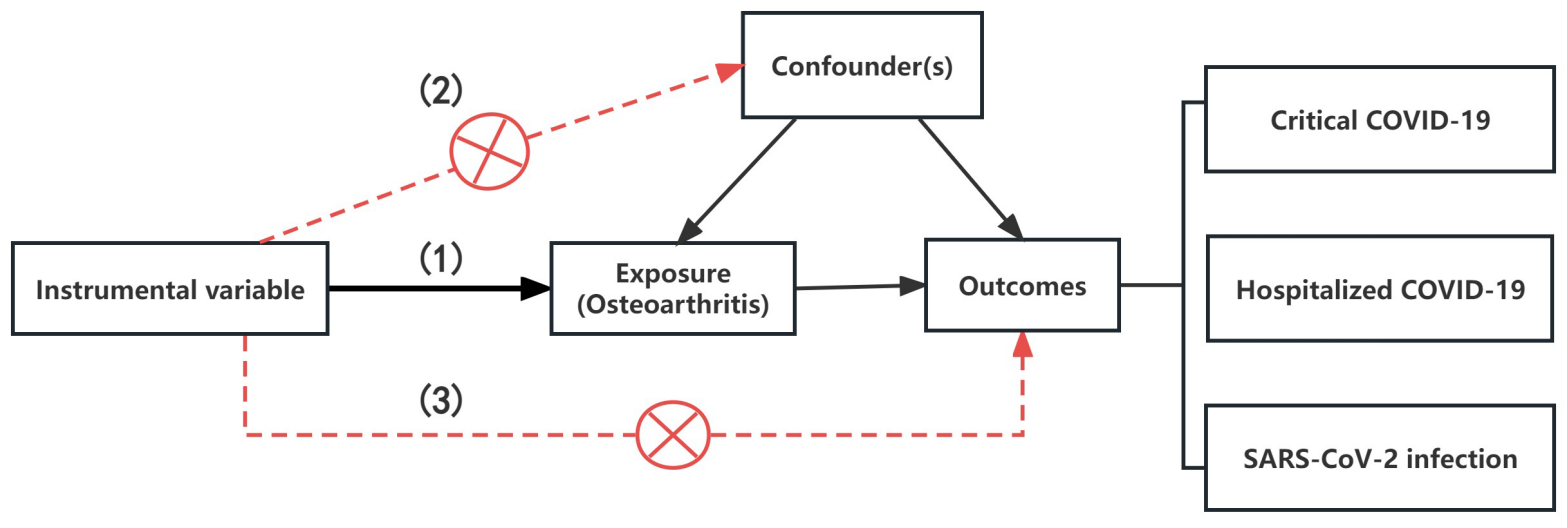
The illustration for the three core assumptions of MR. (1) The IV is associated with the exposure; (2) The IV is not associated with any confounders; (3) The IV affects the outcome only through the exposure and not through any other pathways.

In our analysis, we utilized the R package TwosampleMR (version 0.5.6) to employ MR techniques, including inverse variance weighting (IVW), weighted median (WM), and MR-Egger methods (25). The IVW method, known for its superior statistical power and efficiency when utilizing valid instrumental variables (IVs), elucidates the diversity of causal estimates for particular variables (26). Therefore, we employed IVW as the main approach to calculate the results. To enhance the robustness of our findings, we also employed weighted median (WM) and MR-Egger methods in conjunction with IVW and adjusted the significance level with the false discovery rate (FDR). The instrumental variables (IVs) used for MR analysis must satisfy three core assumptions: (1) the IV is associated with the exposure; (2) the IV is not associated with any confounders; (3) the IV affects the outcome only through the exposure and not through any other pathways (27) (Figure 1).

Single nucleotide polymorphisms (SNPs) were selected as instrumental variables (IVs) from the exposure of interest based on the genome-wide significance threshold (*p* < 5 × 10^−8^), encompassing all variants associated with the exposure. The clumping criterion was set at *r*^2^ < 0.01, within 10 Mb. In sensitivity analysis, we applied the MR-Egger regression test (28) and MR pleiotropy residual sum and outlier test (MR-PRESSO) to assess horizontal pleiotropy and Cochran’s *Q* test (*p* < 0.05) (29) to detect heterogeneity. Furthermore, to test the strong association of a single SNP with exposure and its predominant influence on estimating causal effects, we conducted a leave-one-out analysis (30).

### Knowledge-based analysis

To investigate the molecular links between OA and COVID-19, we conducted large-scale data mining using the PathwayStudio platform^1^ (31) and constructed molecular pathways connecting OA and COVID-19. Initially, we hypothesized that both conditions might share regulatory factors. To validate it, we thoroughly examined relevant references and statements while also ensuring the quality of the extracted relationships by eliminating any irrelevant or indirect connections. Finally, we constructed a network connecting OA and COVID-19 based on the obtained correlations.

## Results

### MR analysis

**Figure 2:**
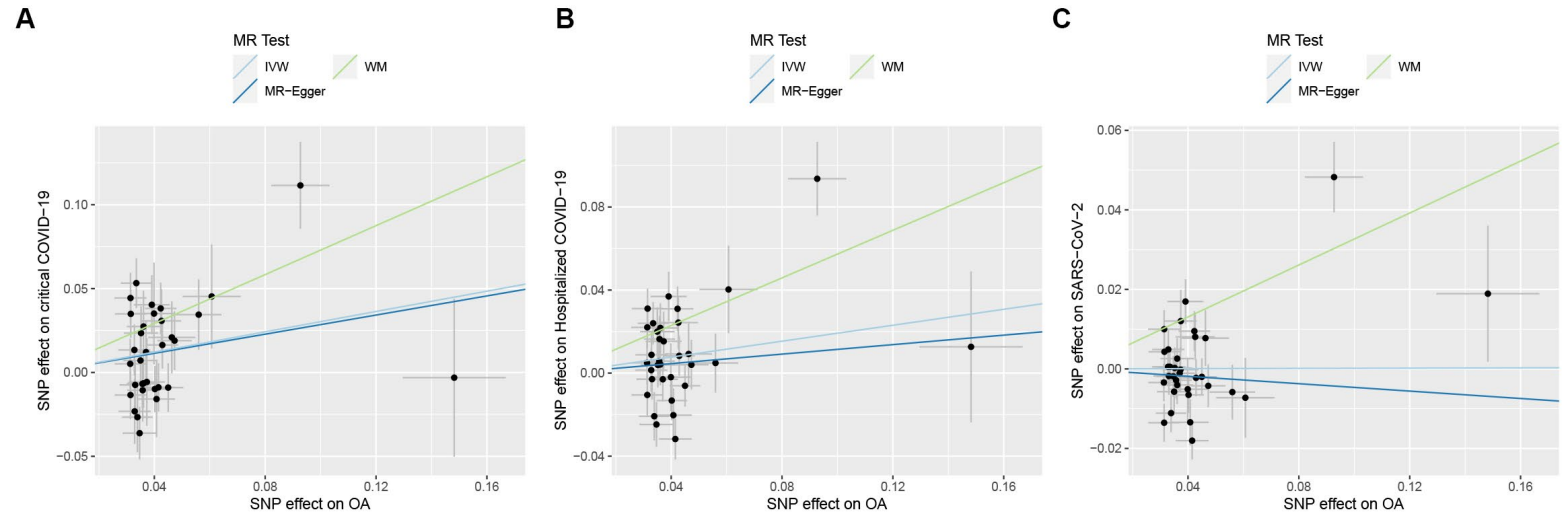
Causal effects of osteoarthritis (OA) on COVID-19 outcomes. **(A)** Causal effects of OA on critical COVID-19 outcome. **(B)** Causal effects of OA on hospitalized COVID-19 outcome. **(C)** Causal effects of OA on SARS-CoV-2 infection outcome. The trait on the *x*-axis denotes the exposure, the trait on the *y*-axis denotes the outcomes, and each cross point represents an instrumental variant. The lines denote the effect sizes of an exposure on an outcome.

In MR analysis, to verify the causality of OA on the three outcomes of COVID-19, a total of 33 genetic variants were obtained as IVs from the OA dataset. According to the IVW method, genetic susceptibility to OA exerts causal influences on hospitalized COVID-19 (odds ratio (OR): 1.21, 95% confidence interval (CI): 1.02–1.43, *p* = 0.026) and critical COVID-19 (OR: 1.35, 95% CI: 1.09–1.68, *p* = 0.006) but not on SARS-CoV-2 infection (OR: 1.00, 95% CI: 0.92–1.08, *p* = 0.969; Table 2 and Figure 2).

**Table 2:** Causal relationships between osteoarthritis and COVID-19 outcomes.

Outcome	Method	B (se)	OR (95%CI)	N_IV	Q_P	P_pleiotropy	*p*	FDR
Critical COVID-19	IVW	0.303 (0.110)	1.35 [1.09–1.68]	33	1.52E-04	NA	0.006	0.036
	MR–Egger	0.729 (0.401)	2.07 [0.94–4.55]	33	1.06E-04	0.278	0.079	0.113
	WM	0.285 (0.120)	1.32 [1.05–1.68]	33	NA	NA	0.017	0.051
Hospitalized COVID-19	IVW	0.192 (0.086)	1.21 [1.02–1.43]	33	6.24E-08	NA	0.026	0.051
	MR–Egger	0.573 (0.333)	1.77 [0.92–3.41]	33	2.62E-08	0.245	0.095	0.114
	WM	0.114 (0.085)	1.12 [0.95–1.32]	33	NA	NA	0.177	0.177
SARS-CoV-2 infection	IVW	0.002 (0.040)	1.00 [0.92–1.08]	33	2.19E-08	NA	0.969	0.969
	MR–Egger	0.327 (0.153)	1.38 [1.03–1.87]	33	1.07E-06	9.52E-05	0.040	0.120
	WM	−0.047 (0.039)	0.95 [0.89–1.03]	33	NA	NA	0.226	0.339

B, effect size; se, standard error; OR, odds ratio; CI, confidence interval; N_IV, number of instrumental variables; Q_P, Cochran’s *p* value of heterogeneity analysis; FDR, false discovery rate; SARS-CoV-2, severe acute respiratory syndrome coronavirus 2; IVW, inverse variance weighted; WM, weighted median; NA, not applicable.

**Figure 3:**
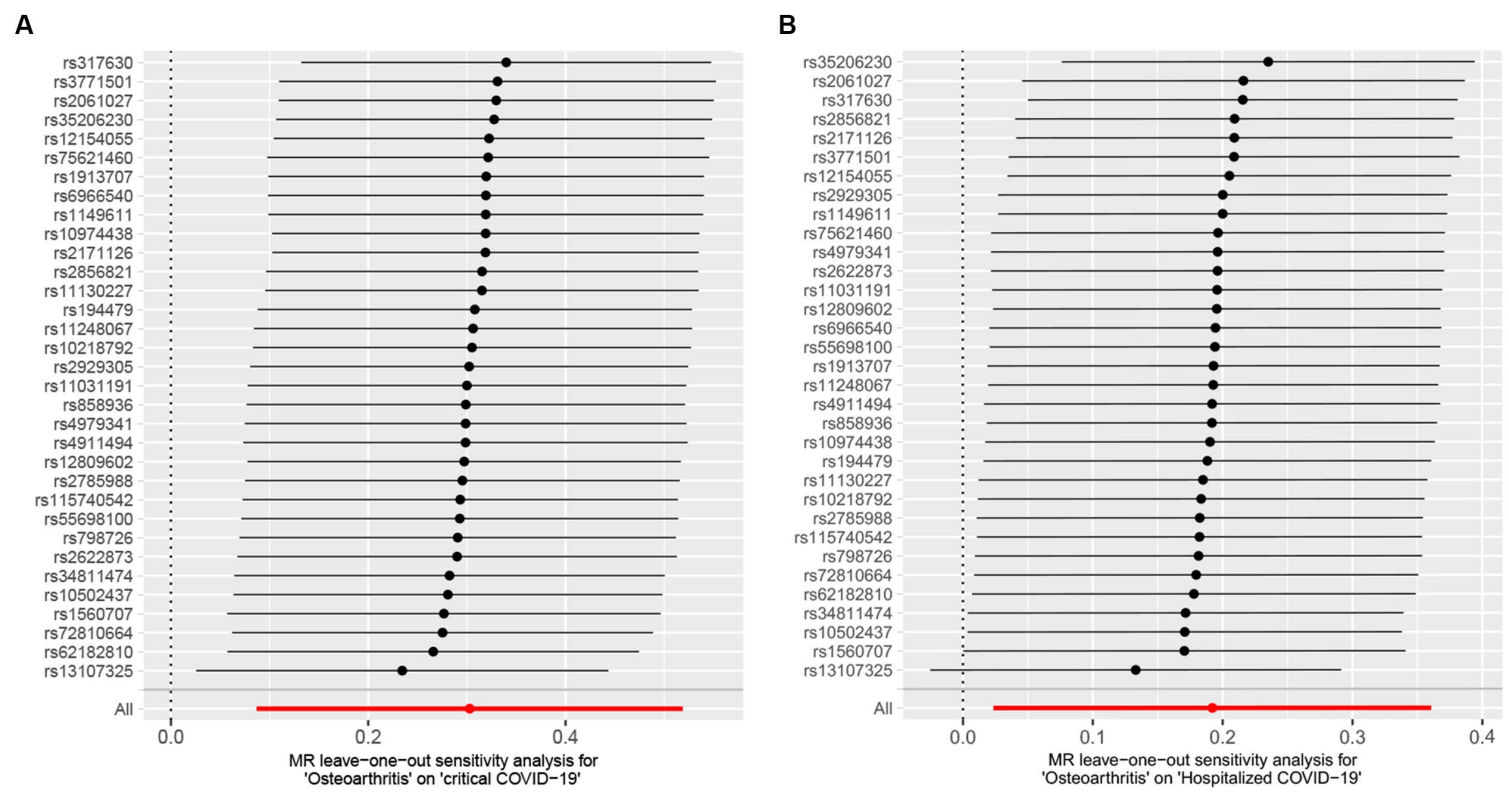
Leave-one-out analysis between osteoarthritis (OA) and COVID-19 outcomes. **(A)** Osteoarthritis on critical COVID-19. **(B)** Osteoarthritis on hospitalized COVID-19.

In sensitivity analyses, MR-PRESSO detected horizontal pleiotropy (*p* < 0.001), while the MR-Egger regression test, which estimates the average horizontal pleiotropy across all SNPs, showed no significant evidence of horizontal pleiotropy (MR-Egger intercept <0.02, *p* > 0.05). Additionally, the FDR test supported the validity of MR results, and Cochran’s *Q* test pointed towards the heterogeneity (*p* < 0.05). On the other hand, the leave-one-out analysis demonstrated that none of the individual SNPs significantly influenced the MR outcomes for severe COVID-19 (Figure 3), which indicates the robustness of the results.

### Knowledge-based analysis

**Figure 4:**
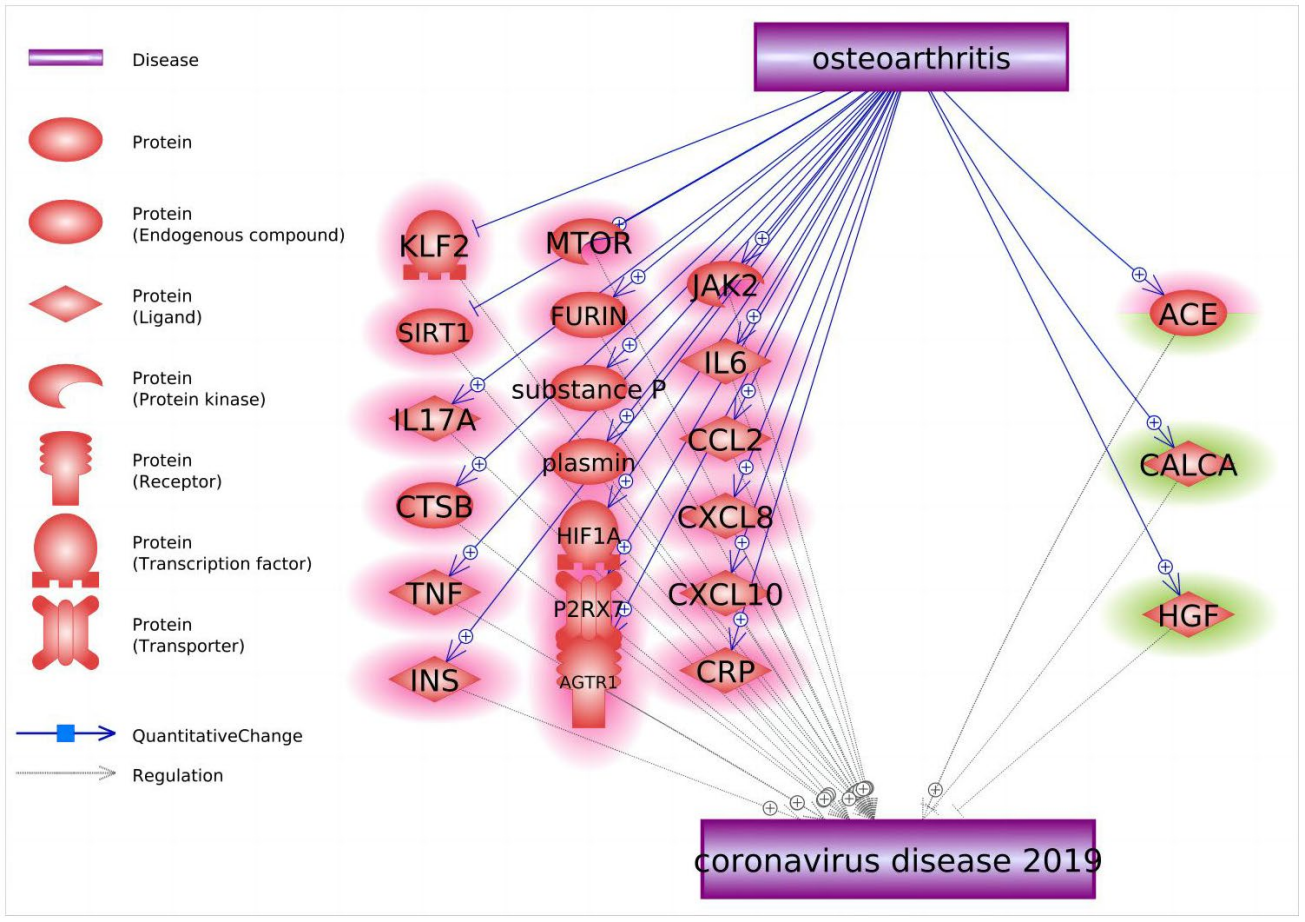
Molecular pathways connecting osteoarthritis and COVID-19. Quantitative genetic changes driven by OA exert more negative (highlighted in red) than positive (highlighted in green) effects on COVID-19.

After inquiring into the molecular relationship between OA and COVID-19, a total of 22 molecular entities were highlighted as candidate mediators of the effect of OA on COVID-19 (Figure 4). Among them, 19 molecules act as negative regulators (*KLF2*, *SIRT1*, *IL17A*, *CTSB*, *TNF*, *INS*, *MTOR*, *FURIN*, substance P, plasmin, *HIF1A*, *P2RX7*, *AGTR1*, *JAK2*, *IL6*, *CCL2*, *CXCL8*, *CXCL10*, and *CRP*), two genes serve as positive regulators (*CALCA* and *HGF*), and one gene, *ACE*, displays bidirectional effects. These 22 molecular players form a network that likely bridges OA and COVID-19.

## Discussion

In contrast to previous studies that mainly reported an intersection of gene sets underpinning susceptibilities to OA and COVID-19 (16–18), our study further elucidated causal genetic associations between these two conditions. The results from our MR analysis show that individuals with OA have a 21% higher probability of hospitalization due to COVID-19 and a 35% increased likelihood of encountering critical COVID-19. Nevertheless, we found no evidence to support a greater risk of contracting SARS-CoV-2 in OA patients. In essence, our findings demonstrate that OA directly influences the severity of COVID-19, contributing to an elevated chance of developing critical conditions, but does not contribute to one’s propropensity of contracting the virus.

The most plausible connection between OA and COVID-19 is through disease-driven alterations in the body’s immune response to inflammation, either due to the presence of the diseases themselves or the effects of medications used to treat them. A majority of the molecules overproduced in patients with OA also contribute to the progression of COVID-19, with only two molecular entities, *HGF* and *CALCA* factors (32, 33), noted as potential suppressors of COVID-19 phenotypes capable of preventing pulmonary fibrosis as well as local and systemic inflammation.

In OA patients, there is often a background elevation of inflammation characterized by increased secretion of IL-6, IL-8, TNF-α, and MCP-1 cytokines, which might, as a group, contribute to the observed association between OA and COVID-19 (34). Crucially, cytokine-producing neutrophils emerge in the synovium right from the initial stages of synovial involvement (35). Consequently, the cytokines and the release of neutrophil elastase advance OA. The same pro-inflammatory proteins secreted by neutrophils are essential for the progression of COVID-19 to its severe form (36). In this manner, the preactivation of neutrophils that is specific to OA could potentially facilitate the progression of COVID-19.

The inflammatory milieux that supports the relationship between OA and COVID-19 may also be further exemplified by related conditions, such as depression. Individuals with physical illnesses commonly co-experience various mental health issues, and depression is one of the frequently observed comorbidities in the aging population. Our previous research suggested that OA and major depressive disorder (MDD) are in a bidirectional causal relationship, which is supported by underlying inflammation and altered estrogen signaling (37). Also, MDD may increase one’s susceptibility to COVID-19, due to pre-existing inflammation (38). Moreover, the close association between MDD and both OA and COVID-19 may serve as a foundation for the observed effectiveness of antidepressant medications in both alleviating pain in OA patients and relieving infection symptoms in individuals with COVID-19 (38, 39). Hence, a role of MDD as a contributing comorbidity or pathophysiological intermediary between OA on COVID-19 should be explored further.

Alternatively, the reasons behind our observations could be attributed to the extended use of NSAIDs and glucocorticosteroids, which is prevalent among OA patients. Both medication groups cause at least some degree of damage to the gastrointestinal lining, either in clinical or subclinical forms (40). In turn, impaired gut barrier function is known to exacerbate virus-induced organ injury, especially when compounded by endotoxemia (7).

The OA and COVID-19 connecting genetic network constructed by a knowledge-based mining approach further clarifies the link between two conditions at the molecular level. First, soluble inflammatory factors such as IL-6, CRP, TNF, and CCL2 are pivotal both for the primary inflammation of OA and for cytokine storms generated by COVID-19 (34, 41–44). Pre-existing elevations in these OA-induced inflammatory factors could exacerbate the cytokine storms of COVID-19, which promotes the deterioration of patients’ condition further. Additionally, other inflammation-related molecules shared between OA and COVID-19, such as these encoded by *HIF1A*, *CXCL10*, and *CTSB* (45–47), contribute to the same pathophysiological bonfire. Of note, elevated levels of IL-17, which is involved in both severe COVID-19 (48) and osteoarthritis (49), is being currently explored as a novel therapeutic target for both conditions (50).

Furthermore, several other, non-inflammatory genes have captured our attention, with one of particular interest being *SIRT1*. The product of this gene regulates a variety of tissue and cellular processes, from apoptosis to muscle differentiation, by catalyzing the deacetylation of its protein targets. A significant role of *SIRT1* is noted in managing oxidative stress and repairing DNA damage (51). Observational studies have shown that the severity of OA is associated with reduced *SIRT1* expression and a decrease in its product in chondrocytes (52). In patients with COVID-19, a decrease in SIRT1 expression is paralleled by elevation of proinflammatory cytokines (53). Therefore, when OA patients are infected with COVID-19, a preexisting state of reduced SIRT1 activity may worsen inflammation and contribute to an increase in mortality risk. It has also been demonstrated that SIRT1 activators, such as cytarabine and resveratrol, may attenuate the development of cytokine storms and alleviate hyperinflammation and neuroinflammation-mediated cognitive dysfunction in COVID-19 patients (54). These SIRT1 activators may be used as adjuvant therapy suitable for patients with OA, with SARS-CoV-2 infection, or with OA/SARS-CoV-2 comorbidity.

ACE plays a two-sided role in the molecular pathways connecting OA to COVID-19. It is well known that OA is associated with an imbalance in ACE/ACE2 expression, with elevation of ACE and concomitant reduction of ACE2 expression (55, 56). When ACE/ACE2 imbalance occurs in the lungs, levels of AngII increase and over-activate pulmonary AT1R, leading to pulmonary capillary permeability. In turn, leaky capillaries cause pulmonary edema, which, when combined with an increased inflammatory response and apoptosis, greatly accelerates lung injury (55, 57). Therefore, alteration of the ACE/ACE2 ratio may aggravate COVID-19 symptoms. Notably, preinfection administration of AT1R blockers significantly lowers mortality of patients with COVID-19 (58), which indicates favorable implications for the treatment of OA patients during the COVID-19 situation.

We have also noticed synergistic OA- and COVID-19-promoting effects of *MTOR*, a major negative regulator of autophagy. *MTOR* contributes to increased chondrocyte apoptosis and cartilage degradation (59). Some clinical studies suggest that inhibition of MTOR may prevent or delay the induction of senescence in OA (60), and that the blocking of MTOR by sirolimus should improve the clinical outcome of SARS-CoV-2 infection (61). It seems that suppression of MTOR may be a promising approach for the concurrent treatment of OA and COVID-19.

While our study effectively elucidates the causal links between OA and COVID-19, certain limitations should be acknowledged. Firstly, the datasets used in our MR analysis include only a population of European ancestry, and therefore, its conclusions should be applied to the overall population with some caution. Secondly, MR studies do not account for the potential impact of environmental factors, and, therefore, we should stress the importance of conducting future observational studies. As MR-Egger and other sensitivity analyses supported the robustness of the results and the IVW method balanced the potential heterogeneity, we deemed our results as reliable. Nevertheless, one should keep in mind indications of pleiotropy and heterogeneity revealed by the MR-PRESSO and Cochran’s *Q* tests.

## Conclusion

Our study strengthens the evidence that OA is causally associated with severe outcomes of COVID-19. This finding informs preventative measures in elderly individuals affected by OA and provides some novel synergistic options for the treatment of SARS-CoV-2 and OA as comorbid conditions.

## Data availability statement

The original contributions presented in the study are included in the article/supplementary material, further inquiries can be directed to the corresponding author.

## Author contributions

LF: Formal analysis, Software, Visualization, Writing – original draft, Writing – review & editing. AB: Writing – original draft, Writing – review & editing. HC: Writing – original draft, Writing – review & editing. FZ: Conceptualization, Methodology, Project administration, Supervision, Writing – original draft, Writing – review & editing.

## Footnotes

^1^ www.pathwaystudio.com
